# Envy and Environmental Decision Making: The Mediating Role of Self-Control

**DOI:** 10.3390/ijerph19020639

**Published:** 2022-01-06

**Authors:** Xinni Wei, Feng Yu

**Affiliations:** Department of Psychology, School of Philosophy, Wuhan University, Wuhan 430072, China; suzy_wei@whu.edu.cn

**Keywords:** episodic envy, dispositional envy, pro-environmental behavior, self-control

## Abstract

Emotions have strong impacts on decision making, yet research on the association between social interpersonal emotion and environmental decisions is limited. The present study uses experimental manipulation and cross-sectional investigation to examine how envy state and personality trait envy influence environmental actions. In Study 1, participants were manipulated to elicit benign and malicious envy, and it was found that benign envy acts as an antecedent of pro-environmental behavior, while malicious envy could contribute to behavior harmful to the environment. Study 2 replicated the results of Study 1 and examined the mediator of self-control through a correlational study. Consequently, people who are high in malicious envy tend to engage in more environmentally harmful activities rather than living a sustainable life, while dispositional benign envy could significantly predict pro-environmental behavior. Moreover, the link between dispositional malicious envy and environmental behavior can be explained by trait self-control, while the mediating effect was silent in dispositional benign envy. The findings shed new light on the impact of social interpersonal emotion on making environmental decisions and its related psychological mechanisms.

## 1. Introduction

Undoubtedly, environmental problems, for example, global warming, resource shortages, air pollution, climate change, and reduction in biodiversity have obtained worldwide attention. Some researchers have argued that many problems are caused by human behavior [1,2]. Therefore, environmental decisions are not just choices of personal lifestyle; they are more related to moral action [3,4]. Pro-environmental behavior (PEB), which consists of recycling water, paper and clothes, conserving energy, taking public transportation, green consumption, eating more fruits and vegetables, and donating to environmental campaigns or charities, is motivated by moral emotions [5,6,7]. Therefore, engaging in sustainable activities facilitate individuals to leave or maintain a good impression and seek high social status [8,9]. Given that pro-environmental behavior requires taking others into consideration, it is equivalent to pro-social moral actions rather than self-directed actions in social contexts [7,10].

Past studies have revealed that factors like personality, environmental knowledge, attitude, and emotion are of great importance in encouraging the engagement of environmentally friendly behavior [11,12]. However, only limited social psychology research has directedly investigated the impact of emotions on the decision-making of pro-environmental behavior [13,14]. More specifically, most previous studies explored the association of environment induced emotions (e.g., pride, fear, and guilt) and pro-environmental behavior [15] but few of them examined the relationship of individuals’ emotional experience and environmental actions [16]. Therefore, the current study aims to investigate how social interpersonal emotion affects environmental decision-making.

### 1.1. Envy and Environmental Behavior

It is well known that social comparisons motivate consumption, including seeking status, showing off personal distinction, and “keeping up with joneses” [17]. As a result, it might contribute to irrational consequences such as overconsumption, resource wasting, and environmental damage [5] Envy occurs in the upward social comparisons when similar people acquire something (e.g., ideal quality, opportunity, task performance and product) that individuals are longing for [18,19]. It is not only defined as a state driven by situations but also conceptualized as a personality trait—dispositional envy that describes the chronic feeling of envy in social settings [20,21]. Based on the dual approach of envy, it is considered as benign envy and malicious envy that entail constructive and destructive aspects [22,23]. The key dimensions that determine what kind of envy appears are the appraisal about the deservingness of envied person’s advantage and the perceived control over the situation of envious individuals [24]. For example, when the advantage held by the superior is appraised as undeserved and the situation is perceived as hard to control, then malicious results are more likely to appear [25].

To alleviate the pain caused by unfavorable social comparison, benign envy might motivate people to improve themselves and to buy the same products that envied people possess, while malicious envy instigates envious individuals to level others down by buying other luxury products that superior does not own [26]. Zheng et al. [27] found that after making upward social comparison, individuals are more likely to spend money on material goods, and both benign and malicious envy act as mediators encouraging engagement in compensatory consumer behavior. That is to say, the more individuals envy others, the more impulsive they would be in consumption. Upward comparisons would result in a high level of materialism, with increased impulsive material consumption [28]. Moreover, much evidence documents that the moral emotion of envy contributes to morally questionable behavioral inclinations [17,29].

The traditional scapegoating hypothesis indicates that people tend to blame a weak target to displace aggression or hatred against the actual and more powerful cause agent because of potential punishment [30,31]. Typically, scapegoating is defined as the act of attributing inordinate blame and punishment to a target for a negative outcome that results from other causes, thus maintaining or restoring personal perceived control over the external world [32]. To illustrate, by considering a target as immoral or by sabotaging and punishing the targeted individuals or groups, people find ways to symbolically eliminate their feelings of inferiority and self-hatred [33]. Therefore, scapegoating serves as a tool to explain failure or misdeed, as well as a method of self-enhancement [34]. From this perspective, it is possible that malicious envy would result in more environmentally harmful behavior than benign envy, such as littering, using more resources, and buying extra and unnecessary products, to alleviate the painful feeling of inferior and negative self-evaluation. 

Despite benign envy also being painful, it is free of hostile inclinations and closely correlated with improving personal status through one’s own efforts rather than harming others [20,22]. To seek high social status and self-improvement, benign envy biases are directed more toward desired advantages than envied persons [35]. Using more sustainable energy and materials is related to high status and distinct preference, which provides envious individuals an alternative approach to display unique taste and improve themselves [8,36]. At the same time, choosing an eco-friendly lifestyle and practicing pro-environmental behavior is novel and unusual, which calls for broad attention and certain self-strength [37]. Since benign envy is more associated with biased attentional focus on self-improvement, it is more likely to motivate people to widen their thoughts and actions that come to mind [38,39]. Hence, by living in an environmentally friendly way, benign envy leads the way to achieve goals.

### 1.2. The Role of Self-Control

Self-control is defined as the capacity that makes people focus on the target and override the conflicts of internal and external desires, thus pushing people to meet moral standards and social expectations [40]. The ability to exert self-control differs within individuals termed as possessing trait self-control [41]. People who are good at self-control are better at inhibiting their impulses and managing self-interested and pro-social conflicts, which is beneficial for performing pro-environmental behavior [42]. More specifically, sense of control has been examined as an important antecedent for predicting pro-environmental behavior, as it is associated with greater willingness to purchase green products, recycling, conserving energy, and seeking and making better use of environmental knowledge [6,43].

In addition, control over the situation is considered a key factor that contributes benign and malicious envy [20,22,24]. If people perceive a high degree of control over the situation, they might benignly envy others and thus less likely to engage in harmful actions [20,24]. However, envy is not a socially desirable emotion, so individuals often hide their authentic feelings by expressing anger or social isolation [44]. To regulate negative emotion and inhibit natural impulses, envy consumes self-control and contribute ego depletion [45]. Consequently, inadequate self-control leads to more immoral and impulsive behavior, including overspending and behaving less pro-socially [46]. Therefore, we assume that self-control acts as a mediator in the association of envy and environmental behavior, including pro-environmental and environmentally harmful behavior. 

In sum, we tested the following hypotheses: (1) Benign envy is positively correlated with pro-environmental behavior but negatively related to environmentally harmful behavior. (2) There is a negative relationship between malicious envy and pro-environmental behavior but a positive relationship between malicious envy and harmful environmental behavior. (3) Self-control mediates the association of envy and environmental decisions.

### 1.3. The Current Research

We conducted two studies to investigate how envy affects environmental decision- making and to examine the role of self-control as the associated mechanism for the effect of envy. Study 1 used an experimental manipulation to test whether episodic envy would influence the engagement of environmentally friendly and harmful behavior. More specifically, it examined whether benign envy would promote more pro-environmental behavior and whether malicious envy would induce more environmentally harmful behavior. To replicate the result, Study 2 used correlational design to explore how the tendency to experience the chronic feelings of benign and malicious envy, dispositional envy, impacts environmental behavior and to examine the potential mediating role of self-control of dispositional envy.

## 2. Study 1: Episodic Envy and Environmental Decision Making

The first study was designed to explore the potential impact of episodic envy on environmental actions. Based on the literature review, we predicted that people would engage in more pro-environmental activities, like recycling, when they are in the benign envy state. In comparison, we assume that malicious envy would motivate individuals to narrow their attention toward negative feelings and less concern the moral principles when making environmental decisions.

### 2.1. Materials and Methods

#### 2.1.1. Participants and Procedure

Ninety-three undergraduate students studying in a college (*M*age = 19.32, *SD* =1.25; 30 male and 63 female) were randomly recruited from psychology classes to take part in this study. After providing informed consent, participants voluntarily joined the study and received course credit as a reward. Initially, all the participants were told that this study was aimed at investigating what kind of life events would make people feel envy. Then, they were randomly assigned to either the malicious envy (N = 52) or benign envy (N = 41) condition. Participants were asked to complete a writing task that manipulated different emotions. In the benign envy condition, participants were told to think about past experiences during which they were envious of others who gained success or advantages through hard work or their own effort. Students under the malicious envy condition were also told that the purpose of the study was to learn which life experiences that could make people feel envious of similar people. However, they were asked to recall a moment that they felt envy because they perceived the advantages of superiors to be undeserved. In both conditions, participants were given 10 min and were required to write down their experiences in detail to describe what happened and how it made them feel at the time. This manipulation was adopted from previous research indicating that the perceived deservingness is a key to distinguishing malicious envy and benign envy [47,48].

#### 2.1.2. Measures

After completing the writing task, all the participants reported their likelihood to engage in environmental actions, including pro-environmental behavior and environmentally harmful behavior [49]. As a measure of pro-environmental behavior (α = 0.70) respondents were asked to report how often they would engage in the following activities: “save energy in the home, plant trees, eat fewer meat meals, recycle, conserve water, use public transport whenever possible, write a letter or sign a petition that is about protecting the environment, buy things that have less throw-away packing”. For the measure of environmentally harmful behavior (α = 0.60), it contained “throw away things that could be recycled, use too much paper, litter, use more water than I should, use more power than I need, and buy things that I don’t need”. Respondents were required to answer these questions from 1 (never) to 5 (always).

Next, participants were asked to indicate whether or not they thought the described person deserved their advantages (1 = not deserving at all, 9 = very deserving) and how much they felt envy (1 = not at all envious, 9 = very envious) at present.

### 2.2. Results

#### 2.2.1. Manipulation Check

For the manipulation check, a one-way ANOVA showed that participants in the malicious conditions (*M* malicious = 4.83, *SD* = 2.05) felt more envy than those in the benign envy condition (*M* benign = 4.29, *SD* = 1.89), although they did not significantly differ (*F* (1, 91) = 1.67, *p* > 0.05). More importantly, consistent with previous studies, participants in the benign envy condition (*M* benign = 7.22, *SD* = 1.92) considered the advantage held by envied individuals to be more deserved compared with the malicious envy condition (*M* malicious = 5.77, *SD* = 2.37), *F* (1, 91) = 10.10, *p* < 0.01, Cohen’s *d* = 0.67, 95% CI [0.24, 1.08]. These results verify the efficacy of our envy manipulation.

#### 2.2.2. The Effect of Envy on Pro-Environmental Behavior

A 2(envy) × 2(environmental behavior) repeated ANOVA showed that there was a main effect of environmental behavior, *F* (1, 91) = 41.56, *p* < 0.001, partial η^2^ = 0.31. However, the main effect of envy was not significant (*F* < 1, *p* = 0.59). There was a significant interaction between envy state and environmental behavior, *F* (1, 91) = 10.94, *p* = 0.001, partial η^2^ = 0.11). Specifically, individuals were more likely to engage in pro-environmental activities in the benign condition (*M* = 3.33, *SD* = 0.52) than in the malicious envy state (*M* = 3.02, *SD* = 0.57), *F* (1, 91) = 7.53, *p* = 0.01, partial η^2^ = 0.08. However, people indicated more possibilities to take part in environmentally harmful behavior in the malicious envy condition (*M* = 2.75, *SD* = 0.42) than in the benign condition (*M* = 2.51, *SD* = 0.57), *F* (1, 91) = 5.65, *p* = 0.02, partial η^2^ = 0.06.

### 2.3. Discussion

Overall, we found support for the notion that envy triggers different environmental outcomes based on its dual approach. Moreover, we discovered that people are more likely to engage in environmentally friendly activities when they feel benign envy and want to keep up with others in a rational way. However, if they felt the advantage held by superiors was undeserved, the malicious envy would induce them to behave more environmentally harmfully. This study examined the role of social interpersonal emotions in making environmental decisions in daily life, echoing with broaden-and-build theory [38,39], as well as expanding on past studies relating to emotion and pro-environmental behavior in social psychology [14,17,50].

## 3. Study 2: Dispositional Envy and Environmental Decision Making

In Study 1, we examined whether people’s episodic envy experience in social contexts was associated with environmental decisions. Consequently, it revealed that benign envy promotes pro-environmental behavior, while malicious envy is more related to harmful actions to the environment. As a socially unacceptable emotion, envy is often hidden or replaced with other feelings, like anger and resentment, because people are reluctant to admit the authentic feeling of envy to others and themselves [24]. Envy is also known as a typical personality trait termed dispositional envy, which describes the chronic tendency to feel envy in social settings [20].

Past studies imply that relatively stable personality traits are predictors of pro-environmental behavior, for instance, social dominance orientation, pro-social value orientation, openness, agreeableness, and neuroticism [11,37]. Borden and Francis [51] have also illustrated that compared with altruism, people who are competitively oriented are less likely to act ecologically. Therefore, we investigated how people who frequently make upward social comparisons and suffer from envy make environmental decisions. To replicate the results in Study 1 and test the mediator of self-control, we recruited participants to complete a cross-sectional study.

### 3.1. Materials and Methods

#### 3.1.1. Participants

Using a convenient sampling method, 257 Chinese college students were selected to voluntarily participate in this study in exchange for partial course credit. Ultimately, 170 participants finished all the items and passed attention check items online, among whom 68 were males and 102 females (*M* age = 20.76, *SD* = 3.55).

#### 3.1.2. Materials

Lange and Crusius [20] developed the benign and malicious envy scale (BeMas) to measure two types of dispositional envy. Dispositional benign envy (e.g., “Envying others motivates me to accomplish my goals”, α = 0.81) and malicious envy (e.g., “Seeing other people’s achievements makes me resent them”, α = 0.85) were assessed with 10 items. Participants answered questions on a 7-point Likert scale ranging from 1 (strongly disagree) to 7 (strongly agree). The overall alpha coefficient for the benign and malicious envy scale was 0.79.

To assess the trait self-control, the Brief Self-Control Scale [41], composed of 13 items (e.g., “I have a hard time breaking bad habits”, “Sometimes I can’t stop myself from doing something, even if I know it is wrong.”) was employed in this investigation. Participants responded on a 5-poiny Likert scale from 1 (not at all) to 5 (very much). The Cronbach’s alpha coefficient of the scale used in this study was 0.84.

Environmental actions including pro-environmental behavior and environmentally harmful behavior were the same as in Study 1. However, participants were asked to indicate the current frequency of behaviors relating to the environment.

Covariates included gender (male = 1, female = 2), region (urban areas = 1, rural areas = 2), and age.

### 3.2. Statistical Analysis

The data were used to conduct the descriptive analysis, correlation analysis, and mediation analysis using SPSS 26 and PROCESS 3.3 developed by Hayes [52].

### 3.3. Results

#### 3.3.1. Descriptive Analysis

Table 1 shows the results of the descriptive analysis of the main variables. Dispositional malicious envy was significantly and negatively correlated with pro-environmental behavior (*r* = −0.18, *p* < 0.05) but positively related to environmentally harmful behavior (*r* = 0.33, *p* < 0.01). However, dispositional benign envy was significantly and positively associated with pro-environmental behavior (*r* = 0.18, *p* < 0.05) and negatively related to environmentally harmful behavior (*r* = −0.13, *p* > 0.05). The correlations of self-control and main variables were all significant, except for dispositional benign envy.

#### 3.3.2. Mediating Effect of Self-Control between Dispositional Benign Envy and Environmental Behavior

To further examine the mediator of self-control between dispositional envy and environmental behavior, we used PROCESS 3.3 to run the mediation analysis.

Firstly, we focused on how self-control influences the association between dispositional benign envy and environmentally harmful behavior. The results showed that dispositional benign envy did not predict environmentally harmful behavior (*β* = −0.13, *t* = −1.70, *p* > 0.05) and self-control (*β* = 0.12, *t* = 1.63, *p* > 0.05). in model 4, dispositional benign envy could not predict environmentally harmful behavior (*β* = −0.08, *t* = −1.15, *p* > 0.05), but self-control was able to significantly and negatively influence environmentally harmful behavior (*β* = −0.39, *t* = −5.44, *p* < 0.001). Moreover, inspection of the 95% confidence intervals indicated that the mediating effect of self-control was not significant (effect = −0.03, 95% CI [−0.08, 0.01]), as 0 was included.

While dispositional benign envy could significantly and positively influence pro-environmental behavior (*β* = 0.18, *t* = 2.32, *p* < 0.05), the mediating effect of self-control in the association of dispositional benign envy and pro-environmental behavior was not significant either because 0 was included in the 95% CI [−0.01, 0.06]) of the indirect effect. Taken together, self-control was not a mediator in the relationship between dispositional benign envy and environmental behavior.

#### 3.3.3. Mediating Effect of Self-Control between Dispositional Malicious Envy and Environmental Behavior

**Figure 1:**
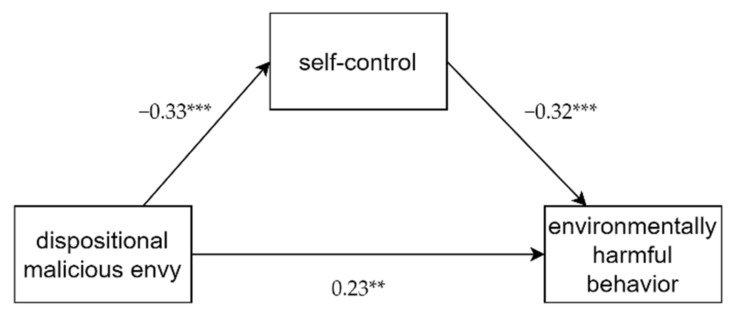
Mediating effect of self-control on DMB and environmentally harmful behavior. *** *p* < 0.001, ** *p* < 0.01.

We also tested whether self-control mediated the relationship between malicious envy and environmental behavior. The results showed that dispositional malicious envy significantly and positively influenced environmentally harmful behavior (*β* = 0.33, *t* = 4.59, *p* < 0.001) and self-control (*β* = −0.33, *t* = −4.46, *p* < 0.001). When placed as predictive variables at the same time, dispositional malicious envy (*β* = 0.23, *t* = 3.13, *p* < 0.01) and self-control (*β* = −0.32, *t* = −4.44, *p* < 0.001) were both able to significantly predict behavior harmful to the environment (see Figure 1). Furthermore, an inspection of the 95% confidence intervals indicated self-control had a significant mediating effect (effect = 0.06, 95% CI [0.02, 0.11]), as 0 was excluded.

**Figure 2:**
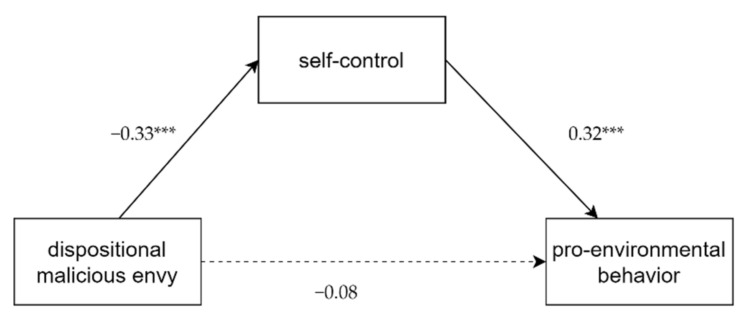
Mediating effect of self-control on DMB and PEB. *** *p* < 0.001.

Additionally, the analysis revealed that dispositional malicious envy significantly predicted pro-environmental behavior (*β* = −0.18, *t* = 2.37, *p* < 0.05) and self-control (*β* = −0.33, *t* = −4.46, *p* < 0.001). When dispositional malicious envy and self-control acted as predictors in model 4, the direct effect of dispositional envy on pro-environmental behavior was not significant (*β* = −0.08, *t* = 0.99). However, dispositional malicious envy was able to negatively influence pro-environmental behavior through the mediator of self-control (*β* = 0.32, *t* = 4.15, *p* < 0.001), as the 95% bias-corrected confidence interval revealed the indirect effect (effect = −0.05, 95% CI [−0.11, −0.01]) was significant (see Figure 2).

Therefore, it manifested that self-control separately mediated the associations of dispositional malicious envy and pro-environmental behavior and environmentally harmful behavior.

### 3.4. Discussion

In this study, we investigated the relationship between dispositional envy and environmental behavior and examined the mediator of self-control. Consistent with our first study, people high in dispositional benign envy are more prone to take part in environmental activities, while individuals with high levels of dispositional malicious envy are more related to environmentally harmful engagement, such as littering and overconsumption. Additionally, trait self-control plays a key role in the association between dispositional malicious envy and environmental behavior, manifesting the importance of self-control in promoting pro-environmental behavior [42,43].

## 4. General Discussion

The results of the two studies support our hypotheses that interpersonal emotional envy is associated with and leads to engagement in environmental behavior. Study 1 indicated that manipulated benign envy acts as a causal predecessor of pro-environmental behavior, while malicious envy can contribute to environmental damage. Consistently, Study 2 evidenced that people who are high in malicious envy tend to engage in more environmentally harmful activities, while dispositional benign envy could significantly predict pro-environmental behavior. Moreover, Study 2 also supported our hypothesis that the link between dispositional malicious envy and environmental behavior can be explained by trait self-control, although the mediating effect was silent in dispositional benign envy.

In Study 1, we manipulated two types of episodic envy—benign envy and malicious envy—which resulted in different environmental consequences. When participants felt benign envy, they were motivated to search for various means of improving themselves or protect their image in the unfavorable upward social comparison [14,22]. Pro-environmental behavior, which is a subset of pro-social behavior, is beneficial to individuals, motivating them to leave a good impression and improve their status [53]. Although benign envy is painful, it entails higher appraisals of perceived control and more positive thoughts than hostile and negative feelings of malicious envy [54]. Studies have proven that emotions containing positive and negative valence influence engagement of pro-environmental behavior, such as engaging in green consumption, taking public transportation, and recycling at home [55]. Consistently, the first study showed that with a positive mindset, benign envy allows people to expand their thought and behaviors and adopt new ways of thinking, thus promoting more engagement in the activities of protecting the environment than malicious envy. In contrast, malicious envy induced more environmentally harmful behavior, echoing the dual approach of envy in which malicious envy is related to deconstructive consequences, as it entails more hostile thoughts and tendencies [24].

In Study 2, we examined the relationship between dispositional benign and malicious envy and environmental behavior. The result indicated that dispositional benign envy is positively associated with pro-environmental behavior, while dispositional malicious envy showed a negative correlation with pro-environmental behavior and a positive correlation with environmentally harmful behavior. Previous studies showed that self-control acts as a mediator of the association between negative emotion and pro-environmental behavior [2,11], which is consistent with the result of Study 2. In other words, people who often experience malicious envy would contribute to decreased self-control, thus steering more environmentally harmful behavior [42,56].

Our research provides some theoretical contributions. First, it sheds new light on how personal emotion affects environmental behavior. The multiple causes (e.g., personality, attitude, value, and belief) of pro-environmental behavior have been heatedly discussed, but there has been limited empirical research focused on the link between affect and emotion indirectly related to the environment [13,14]. Based on the broaden-and-built theory, the current research directly reveals that benign envy, which entails more positive thoughts, promotes more pro-social behaviors in protecting the environment, while malicious envy leads to more irrational and immoral behavior that harms the environment [57]. Second, we found the trait self-control plays a key role in the association between dispositional malicious envy and environmental actions, although the mediating effect was missing in dispositional benign envy. That is, negative experiences are stronger than positive ones [58]. As dispositional malicious envy causes ego-depletion, which influences people less to care about moral standards in making pro-environmental decisions [43,59]. According to the results, people high in dispositional malicious envy are more likely to experience anger, inferiority, hatred, and anxiety, contributing to decreased self-control concerning environmentally friendly behavior. Despite being painful, benign envy highlights the desire to improve status rather than damage the advantages of others or level others down [22,24]. As a consequence, benign envy is not necessarily correlated with decreased self-control. Living a sustainable lifestyle and eating healthy signal high status, as pro-environment behavior serves to display personal privilege and status [8]. Thus, dispositional benign envy is significantly and positively associated with pro-environment behavior because using sustainable energy and material has become a means of self-enhancement [8,17].

The findings of this research also have important implications for environmental protection and environmental education in private and public fields. First, individuals should be aware that upward social comparisons could be the antecedent of environmentally harmful behavior, especially when inferiority, hatred, and anger deplete limited self- control. Secondly, malicious motivations are not only harmful to personal mental health but also detrimental to the natural environment and climate change. Therefore, organizations (e.g., companies and schools) and leaders should carefully design competitive award systems to avoid wasting resources and damaging the environment as a result of the frequent experience of malicious envy. Concerning limitations and future directions, environmental behaviors were assessed using self-reporting measures, which might cause doubts of the accuracy and validity of the present study. Besides, we focused specifically on the mediating effect of self-control without considering other possibilities. Thus, multiple-sourced data, other mediators, and moderators should be taken into account in the future.

## 5. Conclusions

The present research initially examines the impact of envy on environmental decision making and provides new insight into the relationship between emotion, personality traits, and pro-environmental behavior. The feeling of malicious envy induced more environmentally harmful behavior, while benign envy promoted more environmentally friendly behavior (Study 1). In addition, the trait self-control played as mediating roles in the association between dispositional malicious envy and environmentally friendly and harmful behavior separately (Study 2). Taken together, the findings demonstrate that daily emotions and stable personalities are closely related to pro-environmental behavior, especially negative emotion and socially undesirable characteristics, such as malicious envy.

## Figures and Tables

**Table 1:** Descriptive data and correlations of the variables.

Variables	*M*	*SD*	1	2	3	4
1 DME	2.65	1.09				
2 DBE	4.76	0.97	0.15 *			
3 Environmentally harmful behavior	2.57	0.59	0.33 **	−0.13		
4 Pro-environmental behavior	3.11	0.56	−0.18 *	0.18 *	−0.52 **	
4 Self-control	2.83	0.52	−0.33 **	0.12	−0.40 ***	0.34 ***

*** *p* < 0.001, ** *p* < 0.01, * *p* < 0.05; DME = dispositional malicious envy, DBE = dispositional benign envy.

## Data Availability

The datasets generated during and/or analyzed during the current study are not publicly available due to the sensitivity of the questions asked to participants but are available from the corre-sponding author on reasonable request.
